# Acquired hepatocerebral degeneration: A case report

**DOI:** 10.1590/S1980-57642012DN06010010

**Published:** 2012

**Authors:** Clarice Listik, Gislaine Cristina Lopes Machado-Porto, Maira Okada de Oliveira, Fábio Henrique de Gobbi Porto

**Affiliations:** 1 Fifth year Medical Student at the University of São Paulo, São Paulo SP, Brazil; 2 MD. Department of Radiology, Hospital A.C. Camargo, São Paulo SP, Brazil; 3 Psych, M.Sc. Behavioral and Cognitive Neurology Unit, Department of Neurology, and Cognitive Disorders Reference Center (CEREDIC), Hospital das Clínicas of the University of São Paulo (HC/USP), São Paulo SP, Brazil; 4 MD, Behavioral and Cognitive Neurology Unit, Department of Neurology, and Cognitive Disorders Reference Center (CEREDIC), Hospital das Clínicas of the University of São Paulo, São Paulo SP, Brazil

**Keywords:** acquired hepatocerebral degeneration, hepatic encephalopathy, liver disease

## Abstract

Acquired hepatocerebral degeneration is an underdiagnosed neurologic syndrome characterized by parkinsonism, ataxia or other movement disorders and by neuropsychiatric and cognitive symptoms. It occurs in patients with chronic liver disease, especially those who develop portosystemic shunting and is often unrecognized as a cause of cognitive decline. Recently, its pathogenesis has been associated with manganese accumulation in basal ganglia and some treatments proposed. The aim of this article was to report a case and discuss some discoveries in connection with the disease.

## Abstract (pt)

Degeneração hepatocerebral adquirida é uma síndrome neurológica subdiagnosticado caracterizada por parkinsonismo, ataxia ou outros distúrbios do movimento e por sintomas neuropsiquiátricos e cognitivos. Ocorre em pacientes com doença hepática crônica, especialmente aqueles que desenvolvem shunts porto-sistêmicos e frequentemente não é reconhecido como uma causa de declínio cognitivo. Recentemente, sua patogênese tem sido associada ao acúmulo de manganês nos gânglios da base e alguns tratamentos foram propostos. O objetivo do artigo foi relatar um caso e discutir algumas descobertas nesta doença.

## INTRODUCTION

Acquired hepatocerebral degeneration (AHD) is a neurologic syndrome characterized by parkinsonism, ataxia or other movement disorders and by neuropsychiatric and cognitive manifestations, in patients with chronic liver disease, especially those who develop portosystemic shunting.^1,2^ It is often under-recognized as a cause of cognitive impairment in patients with liver disease. Recently, its pathogenesis has been associated with metal accumulation in basal ganglia, mainly manganese, increasing the interest of clinicians and researchers in the condition.^3-5^ We report a case of a patient with clinical and radiological characteristics of AHD, who presented with a rapid cognitive decline, discuss its possible underlying mechanisms and treatments currently available.

## CASE REPORT

A 51-year-old man with a history of chronic hepatitis B virus (HBV) infection was seen in our outpatient neurologic clinic for cognitive complaints. Approximately 1 year earlier, he started having trouble driving his truck (he worked as a truck driver), got lost in familiar streets and on one occasion, forgot where he had parked his truck. His wife noted rapid forgetting in conversation, repeatability and difficulties keeping commitments. After a few months, he was fired from his job because of frequent delays. In the last three months he developed psychotic symptoms, mainly visual hallucinations. He claimed to see dancers on the screen of his computer and Indians running around his house. He also presented confabulations; according his wife he told some stories with distortions of the facts. He started to display lack of initiative and emotional blunting. Together with the cognitive complaints, he began to present tremor in both arms and hands, gait instability, sluggish movements and a "shock-like" movement in his arms.

He had been followed by the infectious disease department for chronic HBV infection since 2009 and presented mild hepatic dysfunction (Child-Pugh score^6,7^ of 6 - Class A). He was treated with interferon and tenofovir. Liver biopsy had shown mild cirrhosis.

**Figure 1:**
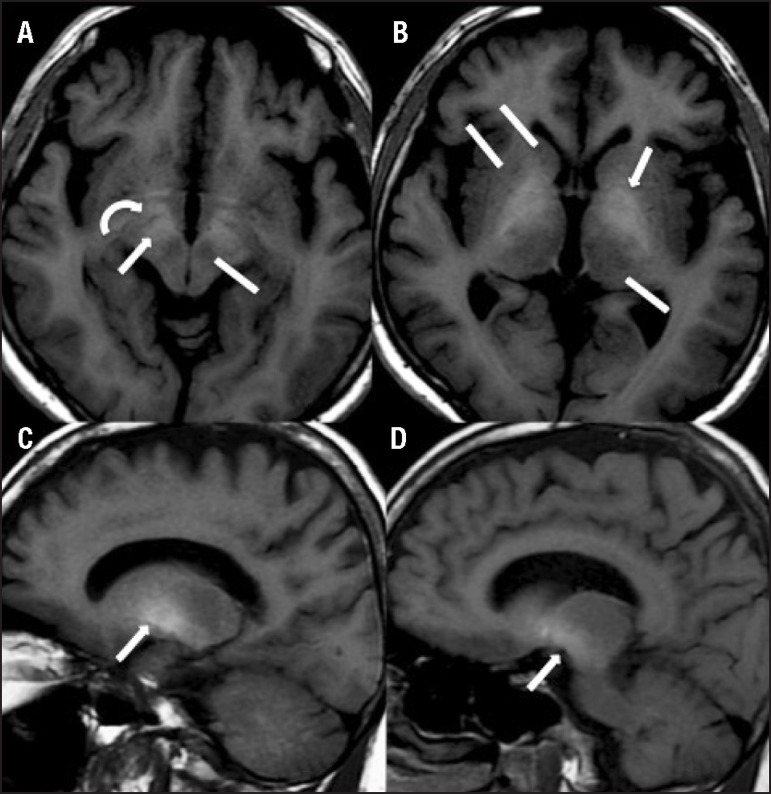
[A,B] Axial T1-weighted magnetic resonance imaging shows symmetrical bilateral hyperintensities involving the substantia nigra (arrow in A), the anterior midbrain (curved arrow in A) and the globus pallidus (arrow in B). The putamen, caudate, thalamus (lines in B) and red nucleus (line in A) are spared. [C,D] Sagittal T1-weighted magnetic resonance imaging also demonstrates hyperintensities in the globus pallidus (arrow in C) and substantia nigra (arrow in D).

**Figure 2:**
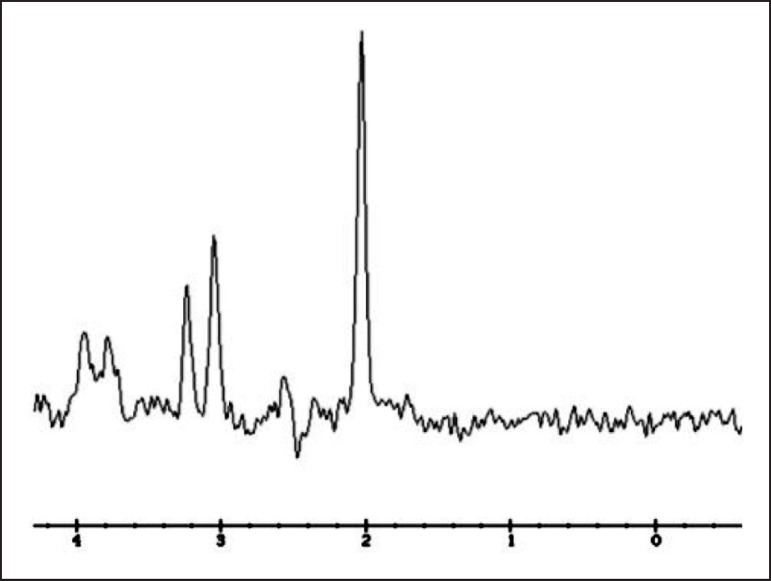
MR-spectroscopy. The main resonance peaks correspond to N-acetylaspartate (NAA, 2.0 ppm), glutamine glutamate (Glx, 2.1-2.5 ppm), creatine phosphocreatine (Cr, 3.02 ppm), choline-containing compounds (Cho, 3.2 ppm), and myo-inositol (Ins, 3.55 ppm). The initial spectrum showed no increase in the glutamate glutamine region and a decrease in the myo-inositol and choline resonances.

Neurologic examination revealed an unsteady gait with lack of associated swing movements, bilateral symmetric rest and postural tremor in arms, mild rigidity and negative myoclonus when his arms were outstretched. He had facial hypomimia and a dysarthric speech. He scored 19/30 on the mini mental state examination (MMSE),^8,9^ with errors in temporal and spatial orientation, immediate memory, calculation, memory recall and language. According to his medical chart, he had scored 30/30 on the same test a year earlier. Neuropsychological evaluation showed severe impairment in motor function (DRS),^10,11^ constructive abilities (Rey Complex Figure copy),^12^ attention (TMA, TMB and Stroop Test),^12^ working memory (digit span),^12^ visual episodic memory (Visual Reproduction -WMS),^13^ auditory-verbal learning (sum total),^14^ and mild to moderate impairment in Initiation/Perseveration (DRS),^10,11^ visual search and reasoning/abstraction,^15^ verbal episodic memory (Logical Memory and Rey Auditory Verbal Learning),^13,14^ verbal fluency (FAS)^12^ and naming by visual confrontation.^12^ Complementary investigation showed elevated serum ammonia (109 µmol/L - reference range: 11-32 µmol/L). The results of serum copper and ceruloplasmin levels and urinary copper were normal. Magnetic resonance imaging (MRI) of the brain showed a mild degree of cortical and subcortical homogeneous atrophy on T1-weighted images, in addition to bilateral and symmetrical hyperintensities in the globus pallidus. Similar hyperintensities on T1-weighted images were found in the substantia nigra, in the ventral aspect of the midbrain and hypothalamus. The putamen, caudate nucleus, thalamus, the red nucleus, and the cerebellum were spared (Figure 1). T2-weighted images disclosed small foci of hyperintensities in subcortical and deep white matter (not shown). MR -spectroscopy showed no anomalous peaks (Figure 2). Abdominal ultrasonography with Color Doppler demonstrated a cirrhotic liver but showed no portosystemic venous shunt.

After discussion, a diagnosis of probable acquired hepatocerebral degeneration was reached. The patient was started on ammonia-decreasing therapy with diet manipulation (diet rich in branched-chain amino acids) and lactulose. Trientine as a potential chelator of manganese was also introduced (long-term outcome unknown at time of writing).

## DISCUSSION

The patient presented with a rapidly progressive cognitive decline associated with neuropsychiatric symptoms and movement disorders. Over the period of one year, his score on the MMSE declined 11 points and he started presenting functional impairment in daily activities. He had no disturbance in awareness that could have explained this finding. Clinical, laboratory and neuroimaging investigations were consistent with the diagnosis of AHD.

AHD is an under-diagnosed neurological condition found in many forms of advanced liver disease (independently of etiology), especially those with portosystemic shunting,^4^ which are either surgically^16^ or spontaneously induced.^17^ This chronic encephalopathy was first reported by van Woerkem in 1914,^18^ but remained unrecognized until 1965 when Victor et al. published their observations.^19^

Persistent chronic liver disease has an estimated 17% prevalence in the general population.^20^ In Brazil, an increase in the prevalence of cirrhosis was observed between 2003 and 2008.^21^ However, the true prevalence of AHD remains uncertain. Nevertheless, Burkhard et al. found that around 21% of cirrhotic patients exhibited parkinsonism, associated or otherwise with other extrapyramidal symptoms.^22^ However, a larger retrospective study found that roughly 1% of patients with cirrhosis had AHD.^23^

AHD is a rare syndrome whose symptoms include ataxia, parkinsonism and other movement disorders,^22^ as well as cognitive dysfunction and neuropsychiatric symptomatology.^24,25^ Psychiatric symptoms may include apathy, lethargy, excessive somnolence, whereas extrapyramidal manifestations range from focal dystonia, postural tremor, akinesia, ataxia, myoclonus, choreoathetosis and others.^2,4,22,25,26^ Although cognitive impairment is part of AHD, it has been overlooked for many years. Patients show attention limitations, particularly with regard to visual-spatial components.^24^

The age of onset is often in the fifth and sixth decades of life,^22,26^ but the disease has also been reported in other age groups, including children.^27^ The condition develops gradually and progressively and the etiology of cirrhosis is not a risk factor for the development of AHD.^22^

The pathogenesis of AHD remains unclear but metal intoxication seems to play a role in the disease. Brain manganese overload^3-5,22,28^ may contribute to an AHD outcome. Some studies suggest that manganese plays a major role in the development of the disease.^22,29^ The hepatobiliary system clears manganese from both blood and cerebrospinal fluid and in some patients manganese concentrations are higher than would be expected. Therefore, the toxic substances that are not removed by the hepatobiliary system due to portosystemic shunts and liver dysfunction enter into systemic circulation.^30^ As a result, manganese deposition in the brain (especially in the basal ganglia, brainstem, cerebral cortex and surrounding white matter) is thought to induce neuronal loss, Alzheimer's type 2 abnormality of astrocytes, among other specific alterations.^29^ This increase in brain manganese has a neurotoxic effect, inducing selective neuronal loss in basal ganglia structures and reactive gliosis.^31^ Studies have shown the toxic effects of manganese to be the major determinant of basal ganglia dysfunction, leading to the predominantly extrapyramidal central nervous system symptoms of cirrhosis.^22^ In the present case, although the patient had ultrasonographic signs of liver cirrhosis and a hepatic biopsy showing mild cirrhosis, he did not present portosystemic venous shunt. However, the presence of liver disease alone, without shunting, is sufficient to cause AHCD. Also, there is a possibility that the shunts are not detectable on ultrasonography, particularly in cases of micro shunts.^31^

Pallidal signal hyperintensity on T1-weighted MRI is a well-described finding in the majority of patients with cirrhosis or portal-systemic shunts, regardless of the etiology of the liver disease.^13,22,31,32^ It is believed that the high signal intensity on T1 is due to the rise in manganese concentration within the CNS, with preferential deposition in the globus pallidus.^22,29^ Manganese is a paramagnetic substance and therefore presents with T1 shortening and can be visualized by unenhanced magnetic resonance imaging. Compounds with paramagnetic properties, such as melanin, methemoglobin and some heavy metals, cause increased intensity of signal limited to T1-weighted images. However, although most patients with cirrhosis present these pallidal hyperintensities on T1-weighted images, not all have neurological symptoms.^16,25^ It has been proposed that the involvement of the substantia nigra on T1-weighted images is a surrogate marker on MRI of parkinsonism symptoms. Burkhard et al. showed that those patients who had hyperintensities not only in the globus pallidus but also in the substantia nigra (some involving the ventral aspect of the midbrain, substantia innominata and hypothalamus) presented with extrapyramidal symptomatology.^22^

In this case, T1-weighted images disclosed bilateral and symmetrical hyperintensities in the globus pallidus and substantia nigra, in the ventral aspect of the midbrain and hypothalamus. The putamen, caudate nucleus, thalamus, the red nucleus, and the cerebellum were spared (Figure 1). These findings support the idea that there is widespread involvement of dopaminergic pathways in basal ganglia circuits.

MR spectroscopy is available for detecting specific biochemical alterations, which consist of increases in cerebral glutamine and glutamate (Glx) and decreases in myoinositol (mI) and choline (Cho) metabolites in chronic hepatic encephalopathy.^33^ In the present case, MR spectroscopy peaks showed no increase in Glx, a typical finding in hepatic encephalopathy. It should be noted that at the time of MRI, the patient was already being treated for reduction of ammonia, and this may explain the absence of the peaks of Glx. However, the persistence of clinical symptoms associated with other MRI findings support the diagnosis of hepatocerebral degeneration as a distinct syndrome of hepatic encephalopathy.

White matter focal lesions (WMLs) on T2-weighted imaging may also be present in patients with liver cirrhosis, with or without overt HE.^31^ Cortical hyperintensities on T2-weighted images correspond to pseudolaminar spongy degeneration in the deep layers of the cerebral cortices while hyperintensities in cerebral white matter correspond to tissue rarefaction associated with loss of myelin and axons but without reactive astrocytosis.^29,34^

The differential diagnosis includes Wilson's disease and hepatic encephalopathy. Wilson's disease presents characteristic Kayser-Fleischer rings, family history, elevated urinary copper excretion, usually reduced serum ceruloplasmin levels, and increased T2 signal in the basal ganglia, white matter, thalamus or brainstem.^2,36^ However, disease differentiation with hepatic encephalopathy can be more challenging.^36^ The presence of concomitant hepatic encephalopathy (even subtly) may overlap with the clinical course of AHD, with hyperammonemia playing a role in the condition.^25^ Reduced level of consciousness and response to ammonia-lowering therapies may be indicative of hepatic encephalopathy. Overall, AHD may be challenging to diagnose because of its rarity, variable clinical presentation and presence of other concomitant conditions.^37,38^

Whether AHD is reversible remains controversial. Liver transplantation may be an effective therapy.^22,25^ Nevertheless, despite the fact that the condition can improve with this procedure, some patients do not respond to this approach^23^ while in others the neurological deficits re-emerge after transplant.^21,29^ Also, treatment with trientine has been reported to successfully reduce clinical symptoms in some reports. In the present case, treatment using both ammonia-lowering therapies and trientine were tried empirically. Given the patient's initial liver disease, liver transplantation was not indicated.

A limitation of this case study was that serum levels of manganese were not available. Nonetheless, in a study by Spahr et al., 88% of cirrhotic patients, regardless of etiology of liver disease, presented with high pallidal signal and 57% had high blood manganese levels.^32^ This suggests that MRI is a sensitive marker of AHD, even in the absence of serum manganese.
